# “Everybody Brush!”: Protocol for a Parallel-Group Randomized Controlled Trial of a Family-Focused Primary Prevention Program With Distribution of Oral Hygiene Products and Education to Increase Frequency of Toothbrushing

**DOI:** 10.2196/resprot.4485

**Published:** 2015-05-22

**Authors:** Joana Cunha-Cruz, Peter Milgrom, R Michael Shirtcliff, Colleen E Huebner, Sharity Ludwig, Gary Allen, JoAnna Scott

**Affiliations:** ^1^Northwest Center to Reduce Oral Health DisparitiesDepartment of Oral Health SciencesUniversity of WashingtonSeattle, WAUnited States; ^2^Advantage Dental Services, LLCRedmond, ORUnited States; ^3^School of Public HealthDepartment of Health ServicesUniversity of WashingtonSeattle, WAUnited States; ^4^School of DentistryDepartment of Pediatric DentistryUniversity of WashingtonSeattle, WAUnited States

**Keywords:** oral hygiene, toothbrushing, dental devices, home care, dental care, communication, social support

## Abstract

**Background:**

Twice daily toothbrushing with fluoridated toothpaste is the most widely advocated preventive strategy for dental caries (tooth decay) and is recommended by professional dental associations. Not all parents, children, or adolescents follow this recommendation. This protocol describes the methods for the implementation and evaluation of a quality improvement health promotion program.

**Objective:**

The objective of the study is to show a theory-informed, evidence-based program to improve twice daily toothbrushing and oral health-related quality of life that may reduce dental caries, dental treatment need, and costs.

**Methods:**

The design is a parallel-group, pragmatic randomized controlled trial. Families of Medicaid-insured children and adolescents within a large dental care organization in central Oregon will participate in the trial (n=21,743). Families will be assigned to one of three groups: a test intervention, an active control, or a passive control condition. The intervention aims to address barriers and support for twice-daily toothbrushing. Families in the test condition will receive toothpaste and toothbrushes by mail for all family members every three months. In addition, they will receive education and social support to encourage toothbrushing via postcards, recorded telephone messages, and an optional participant-initiated telephone helpline. Families in the active control condition will receive the kit of supplies by mail, but no additional instructional information or telephone support. Families assigned to the passive control will be on a waiting list. The primary outcomes are restorative dental care received and, only for children younger than 36 months old at baseline, the frequency of twice-daily toothbrushing. Data will be collected through dental claims records and, for children younger than 36 months old at baseline, parent interviews and clinical exams.

**Results:**

Enrollment of participants and baseline interviews have been completed. Final results are expected in early summer, 2017.

**Conclusions:**

If proven effective, this simple intervention can be sustained by the dental care organization and replicated by other organizations and government.

**Trial Registration:**

Trial Registration: ClinicalTrials.gov NCT02327507; http://clinicaltrials.gov/ct2/show/NCT02327507 (Archived by WebCite at http://www.webcitation.org/6YCIxJSor).

## Introduction

### The Importance of Brushing Teeth Twice a Day

Dental caries is a disease with marked socioeconomic and regional disparities. In Oregon, untreated tooth decay is twice as common in low-income children than in children from higher income families (25% vs 13%) [1]. Similarly, almost three-quarters (73%) of 6 to 9 year old children in rural Central Oregon experience tooth decay, while the statewide average is 52% [1]. Dental caries can be prevented through regular toothbrushing with fluoridated toothpaste [2-5]. The American Academy of Pediatric Dentistry and the American Dental Association recommend that toothbrushing should be performed twice daily with a fluoride toothpaste and a soft toothbrush initially by the parent, and eventually by the child [6,7]. Relatively few parents and children fully follow this recommendation [8].

Successful behavioral interventions focus on helping people acquire skills and motivation to change behavior; they are sustained and comprehensive; and, they engage peers and family members to help maintain motivation [9]. Toothbrushing beginning in infancy is a good candidate behavior. It is similar to other mildly intrusive caregiving behaviors that parents accept learning, and infants grow to tolerate. Parents’ confidence to carry out this behavior is the best predictor of being caries free at age 4 [10,11]. Once established, childhood toothbrushing habits persist [12,13], but need to be reinforced.

Toothbrushing promotion programs that provide advice, free toothpaste, and toothbrushes at a child health visit and/or by mail have shown positive effects on parents’ behaviors, including the frequency of twice daily toothbrushing of their children [14]. When accompanied by social support and in-person instruction, toothbrushing promotion programs show decreases in childhood caries and improved oral hygiene [15-17]. A program similar to that designed for the intervention study described here led to increased frequency of toothbrushing at home [17], and a 30% reduction in tooth decay [16].

### Aims and Objectives

Our aim is to design, deliver, and evaluate the “Everybody Brush!” program, a quality improvement effort of Advantage Dental Services, LLC for children and adolescents enrolled in Medicaid and their families in Central Oregon. Our primary objective is to determine if the intervention is effective in reducing restorative dental care treatment and increasing the percentage of parents who report twice daily toothbrushing of their child’s teeth. This objective reflects key evidence-based recommendations of brushing teeth with fluoride toothpaste (evidence level I) twice a day (evidence level IV). Our secondary objectives are to investigate if the intervention is effective in improving oral health and oral-health related quality of life and reducing dental care costs.

This protocol follows the SPIRIT [18] and Consolidated Standards of Reporting Trials (CONSORT) statements [19] and relevant extensions [20]. The main research question is, “Does the distribution of free toothpaste and toothbrushes with behavioral psychosocial health messages and telephone support, compared with distribution of free toothpaste and toothbrushes alone or no intervention, improve home toothbrushing behavior and reduce the restorative care of children of low-income families?”.

## Methods

### Design, Setting, and Selection of Participants

The study design is a parallel-group randomized controlled trial. The setting is three counties in rural Central Oregon (Crook, Deschutes, Jefferson).

### Eligibility and Recruitment

#### Selection of Participants

The study population will be approximately 20,000 families who are residents of the three county study setting, whose children/members are enrolled in the Oregon Health Plan and served by a single dental care organization. Inclusion criteria will be: (1) children less than 21 years old; (2) enrollment in the public health insurance program, Oregon Health Plan (Medicaid), and served by a single dental care organization, Advantage Dental Services, LLC; and (3) children whose home address is located in the three selected counties.

#### Recruitment

Participants will be identified through an electronic search of the enrollment database of Advantage Dental Services. All Advantage Dental Services members meeting the eligibility criteria will be included. The intervention will be delivered to all children less than 21 years old, but the evaluation of parent-reported twice daily toothbrushing and dental caries outcomes will focus on children less than 36 months old. Advantage Dental Services decided to focus on these children because of the Healthy People 2020 objective OH-1.1 to, "Reduce the proportion of children age 3 to 5 years with dental caries experience", the belief in the lifelong benefit of establishing toothbrushing habits in early childhood, and the cost implications of providing care for children this age who experience tooth decay. Because of all this, a random subsample of parents/caregivers of children less than 36 months old and a random subsample of children less than 36 months old will be recruited. Parents/caregivers of children less than 36 months old to be interviewed will be recruited through telephone calls. Children less than 36 months old to be clinically examined will be recruited at community settings such as Head Start and the Special Supplemental Nutrition Program for Women, Infants, and Children. The parents/caregivers will have the quality improvement project explained to them and have the opportunity to opt out of the evaluation.

The University of Washington Institutional Review Board reviewed this study. Consistent with US Federal regulations, participants of this quality improvement project do not meet the criteria to be considered research subjects. University of Washington personnel will have access only to deidentified data.

### Randomization and Blinding

Families will be randomized with equal probability to one of three study conditions using computer-generated random numbers. A biostatistician at the University of Washington using a dataset containing only family identification codes and without personal identifying information will generate the allocation schedule for random assignment. The treatment allocation for each participant will be kept in Seattle, and the biostatistician will reveal the treatment allocation to local investigators when they are ready to implement the program. Personnel involved in the delivery of the intervention and trial participants will not be blinded to group allocation. Interviewers will be blinded to group allocation.

### Intervention

The goal of the intervention is that parents will brush their young children’s teeth and older children and adults will brush their own teeth twice a day with fluoridated toothpaste. Our strategies to promote this behavior reflect the integrative model of health behavior of Fishbein et al [21] and expansions by Michie et al, Michie et al, and Cane et al [22-24] (Table 1). These guides to the processes that govern health behavior were proven useful in a prior small-group intervention we designed, tested, and found to be successful to increase parents’ frequency of brushing their preschool-age children’s teeth. In that study of 67 families, the intervention components rated most highly by parents were the provision of free toothbrushes and toothpaste for all members of the family, hands-on instruction in how to brush a child’s teeth, and tips to overcome a child’s resistance and make toothbrushing “fun” [25]. In the formative work that led to the intervention’s design, we found parents who brushed their children's teeth twice a day were more likely to describe using specific skills to overcome barriers, have high self-efficacy for toothbrushing, and have high self-standards for establishing it as a routine. In contrast, parents who brushed their children's teeth less than twice daily were more likely to hold negative or false beliefs about the benefits of twice daily toothbrushing, report little normative pressure or social support for the behavior, have lower self-standards, describe more external constraints, and offer fewer ideas to overcome barriers [8].

**Table 1:** Mapping of the components of the “Everybody Brush!” intervention to the theoretical domains, intervention functions, and behavior change techniques.

Intervention component			Theoretical domain	Intervention function	Behavior change technique
**Toothbrushing supplies (kit)**					
	7 toothpastes and 7 toothbrushes		Environmental resources	Enablement	Adding objects to the environment
**Toothbrushing supplies helpline**					
	Request more toothbrushing supplies by telephone		Environmental resources	Enablement	Adding objects to the environment
**Toothbrushing advice helpline**					
	Receive advice on safety, amount, technique, how to overcome barriers and make it fun		Knowledge, physical skills, cognitive skills, beliefs about capabilities, and beliefs about consequences	Education, incentivization, and persuasion	Instruction on how to perform the behavior, information about health consequences, information about emotional consequences, goal setting, problem solving, action planning, restructuring the physical environment, and restructuring the social environment
**Health promotion messages via mail and telephone**					
	**Postcard #0 (cling sheet)**				
		Brush 2 times every day, a tiny smear for baby, a pea size for ages 2 years to adult, use fluoride toothpaste to prevent decay, and you don’t need a lot of toothpaste to make it work	Physical skills, beliefs about consequences	Training, education	Goal setting, instruction on how to perform the behavior
	**Postcard #10 (thank you)**				
		The best way to promote oral health and prevent tooth decay is by brushing teeth with fluoride toothpaste every day two times a day. This simple step has been proven to be effective in promoting oral health and reducing tooth decay and dental care costs.	Knowledge, beliefs about consequences	Education, incentivization, coercion	Goal setting, information about health consequences, credible source, behavior cost
	**Message #1 (brush front and back)**				
		Brush the front and the back of all the teeth. Roar like a lion to reach the back of all the teeth. Say “Chee-tah!” to reach the front and sides.	Physical skills	Training	Instruction on how to perform the behavior
	**Message #2 (take turns)**				
		Take turns brushing... your child practices brushing first and then you do it to make sure all the teeth are brushed.	Cognitive skills	Training	Problem solving
	**Message #3 (sleepy)**				
		Brush in the evening after snacks before your child gets too sleepy.	Cognitive skills	Environmental restructuring	Action planning, restructuring the physical environment
	**Message #4 (bathtub)**				
		You can brush your child's teeth in the tub! It's fun!	Cognitive skills, emotion	Environmental restructuring	Action planning, information about emotional consequences, restructuring the physical environment
	**Message #5 (silly song)**				
		Sing a favorite song while brushing!	Cognitive skills, emotion	Environmental restructuring	Distraction
	**Message #6 (role model)**				
		Brush your teeth with your child. You are the best role model.	Identity	Modeling	Identification as role model
	**Message #7 (party, party)**				
		Have a family toothbrushing party!	Emotion	Persuasion	Restructuring the social environment
	**Message #8 (try, try again)**				
		Everyday is a new day to brush... twice a day!	Beliefs about capabilities, optimism	Persuasion	Verbal persuasion about capability
	**Message #9 (consequences)**				
		Brushing makes your mouth smell fresh.	Emotion, beliefs about consequences	Persuasion	Information about emotional consequences

### Test Condition

#### Components of the Intervention

Participants will receive all five components of the intervention: (1) a toothbrushing kit containing supplies and a single-page instruction sheet sent by mail to families’ homes; (2) a toll-free telephone helpline to request more toothbrushing supplies; (3) postcards with brief messages addressing common barriers and support for toothbrushing; (4) automated calls to parents’ telephones repeating these same health promotion messages; and (5) a toll-free telephone helpline to provide toothbrushing advice. Each of these components was designed in English and Spanish.

#### The Toothbrushing Kit

The kit contains supplies for adults and for children, specifically 7x .85 oz. tubes of toothpaste (1500 parts per million sodium fluoride), 3 adult-size toothbrushes, 1 toothbrush each for a child ages 4-24 months, 2-4 years, and 5-7 years. This quantity was chosen because the average family served by Advantage Dental Services has 3 or 4 children. In addition to the supplies, the kit includes a single-page removable “cling sheet” with instructions to brush twice a day with an age-appropriate amount of fluoride toothpaste depicted by illustration, and information about how to request additional supplies if needed. Each family receives three kits over the 9-month intervention period.

#### Toothbrushing Supplies Helpline

A toll-free number for requesting more toothbrushing supplies is included on all toothbrushing kits mailed to families. Bilingual, culturally competent Advantage Dental Services staff members manage the supplies helpline.

#### Toothbrushing Promotion Message Postcards

We created 9 health messages. The messages reflect three sets of factors known to influence health behavior: strong intention, necessary skills, and lack of insurmountable environmental constraints [21]. The messages are written at a sixth-grade (about 11 years old) reading level and do not contain medical or dental jargon. Each message, and the theoretical domains that inspired it, are in Table 1. As postcards, the messages are accompanied by simple line drawings or pictorial aids to increase visual appeal and parents’ comprehension of the written words [8,26]. An example message, designed to help in restructuring the physical environment, action planning, and to provide information about natural emotional consequences is, “You can brush your child’s teeth in the tub! It’s fun!” The accompanying drawing is of a child in a bubble bath having his teeth brushed by an adult. The postcard messages are mailed semimonthly during the first three months, and then approximately monthly.

#### Toothbrushing Promotion Phone Calls

A local radio celebrity, who identifies himself by his name on the prerecorded telephone message, delivered the health promotion messages described above (Table 1). We chose this strategy to increase perceived social support from a credible source for the intervention goal. The messages were modified for telephone delivery to make them more personable, personal, and to keep them brief. For example, the recommendation, “You can brush your child’s teeth in the tub! It’s fun!” was revised as, “You know, you don’t have to brush your child’s teeth at the sink all the time. Try brushing in the bathtub for something new and fun.” There are eleven telephone message calls (two repeated) that are made, semimonthly during the first three months, and then monthly.

#### Toothbrushing Advice Helpline

A toll-free number for toothbrushing advice is included on all printed materials mailed to families and in the prerecorded telephone messages. Bilingual, culturally competent Advantage Dental Services staff members manage the helpline. The staff have been trained by the University of Washington investigators to be familiar with frequently asked questions about brushing children’s teeth and using fluoridated toothpaste, and how to offer appropriate suggestions to overcome barriers to twice daily brushing.

### Active Control Condition

Participants will receive the toothbrushing kits and access to the telephone helpline for requesting additional supplies, but no additional health messages by postcard or telephone, and no access to the toothbrushing advice helpline.

### Passive Control Condition

Participants will receive the usual Advantage Dental Services practices, which may include receiving information about dental benefits via telephone and mail. This group will be on a waiting list for the trial intervention for 9 months and will then receive one toothbrushing kit. Only children less than 36 months old and their families will be included in this control condition.

By the conclusion of the study, all families will have received toothbrushing supplies. This principle, that all eligible Advantage Dental Services members will have the opportunity to benefit, is part of the organization’s mission.

### Intervention Fidelity

We plan to evaluate the fidelity of the intervention by assessing the extent to which the intervention is delivered as planned. To do so, we will confirm delivery of the toothbrushing supplies, postcards, and prerecorded telephone messages. Participants’ use of the telephone helpline or supply line will be documented, and the nature of these contacts will be analyzed by type of request.

### Study Measures and Data Collection

#### Outcomes

The primary outcome for all children less than 21 years old will be restorative dental care received as a proxy for the presence of dental caries. Additionally, for children less than 36 months old at baseline, the other primary outcome will be parent/caregiver reported frequency of twice daily toothbrushing the child’s teeth. Secondary and tertiary outcomes will be cost of dental care for all participants; and, only for children less than 36 months old at baseline, parent/caregiver-reported oral-health related quality of life of the child, satisfaction with the program, and dental caries (tooth decay).

#### Mediators and Confounders

Mediators of the effect of the intervention on the primary outcomes will be the number of toothbrushing kits and instructions (mail and telephone) delivered. Parent’s age, family size, and race/ethnicity will be considered confounders and explored for effect modification. Among children less than 36 months old, potential mediators to be examined are: parent/caregiver’s self-efficacy, attitudes, intention, skills, and norms in relation to brushing their child’s teeth. Additionally, for this group, parental educational level and child’s juice consumption will be considered confounders.

#### Data Collection

Data on type, amount, and cost of dental care will be collected through health information systems of the dental care organization (Enrollment and Claims Database) for all participants. For children less than 36 months old, information on the children’s toothbrushing behaviors, parent-rated oral health and oral health-related quality of life of the child, child’s juice consumption and frequency, parental self-efficacy, intention and attitudes regarding brushing the child’s teeth, parent educational level, and their opinions about each component of the intervention will be collected through telephone interviews with parents/caregivers at baseline and at the end of the intervention. For children less than 36 months old at baseline, information on the presence of untreated dental caries (cavitated caries in permanent or primary teeth) will be collected through a clinical examination 24 months after the study start date. Fidelity information will be obtained through examination of internal records of mailings, returned mail receipts, and telephone calls.

### Statistical Analysis

#### Sample Size

Sample size for dental care outcomes was not calculated, as the sample size of 20,000 participants was deemed sufficient to observe an effect of the intervention on the primary outcome of restorative care utilization.

Prevalence and effect size estimates for frequency of twice daily toothbrushing among children less than 36 months old at baseline was based on that reported in a study in a similar population [25]. Assuming a proportion in the passive control group of .6, alpha = .025, beta = .20, the required sample sizes for effect sizes of 50% and 40% are 39 and 66 participants in each group. We decided on a sample size of 150 participants in each group.

Untreated dental caries estimate was based on that reported in a study in a similar population [1]. Assuming a proportion of children with dental caries experience in the passive control group of .5, alpha = .025, beta = .20, the required sample sizes for effect sizes of 30% and 40% are 206 and 113. We decided on a sample size of 210 participants in each group.

#### Statistical Analysis Plan

Descriptive statistics (means, SD, counts, and percentages) will be calculated for all variables of interest overall and stratified by age group. Difference in difference models will be used to evaluate changes in frequencies and rates for pre versus post intervention effects within the intervention groups and for passive control versus active control versus test effects. Linear regression will be used to evaluate continuous outcomes and logistic regression will be used to evaluate binary outcomes. The primary hypothesis for all participants is that the test intervention group will have a lower number of restorative dental care procedures received than the active control group during the 18 months post intervention start date. For children less than 36 months old, the primary hypothesis is that the test intervention will increase toothbrushing frequency more than the active control intervention and no change in passive control group will be observed 10 months post intervention start date. Secondary hypotheses are that member satisfaction and oral health-related quality of life of the participants will be greater and that dental care costs and dental caries will be lower in the test intervention and active control than in the passive control.

### Data Management and Quality Assurance

Advantage Dental Services staff members will collect the data. Interview responses will be entered in a secure and US Health Information Portability and Accountability Act of 1996 compliant database with range checks for data values.

### Ethics and Dissemination

The results from the trial will be published regardless of the outcome. Reporting of this trial will adhere to the relevant and most up-to-date CONSORT statement [19] and its relevant extensions [20]. The investigators will ensure that the trial is conducted in compliance with this protocol and federal regulations.

## Results

### Timing of Recruitment, Intervention Delivery, and Follow-Up

Participants were recruited in June 2014, and the intervention is being delivered from August 2014 through April 2015. The toothbrushing supply kits were sent in August and November 2014 and February 2015. Postcards and telephone messages are being delivered to the test intervention group from August 2014 through April 2015. The helpline and other telephone services to request additional supplies are available throughout the intervention period, from August 2014 through April 2015.

Baseline interviews with the subsample of 450 parents/caregivers of children less than 3 years old have been conducted in June and July 2014, and final interviews will be conducted in May 2015. Dental claims data for all participants will be extracted in August 2015. Clinical examination of the subsample will be conducted in July 2016.

### Trial Status

Participants were selected (N=21,743 families, 2857 with children less than 36 months old at baseline) and randomly assigned to the test (n=10,797), active control (n=10,796), and passive control (n=150 families with children less than 36 months old) conditions. A random sample of parents/caregivers of children less than 36 months old was interviewed (n=450, 150 for each test, active, and passive control conditions). The program has been deployed.

## Discussion

The 9-month intervention described in this report, “Everybody Brush!”, builds on our previous work and presents a sizeable challenge to promote twice daily toothbrushing and test the effectiveness and acceptability of health promotion strategies to reach thousands of children and their families. It is a quality improvement project designed to shift the allocation of resources of a dental care organization from restorative dental services to preventive home care practices, specifically toothbrushing with fluoridated toothpaste. The focus of the evaluation on the oral health of the youngest members is because toothbrushing habits established during childhood persist [12,13], and because of the high costs associated with care of young children with rampant dental caries.

The program is being delivered as a universal preventive intervention to all eligible members served by the dental care organization within the three county study setting. There are pros and cons to this decision. A benefit of a universal approach is that it can build wide community recognition and public support for the program. A disadvantage is that it is more costly than including only families with children perceived to be at high risk for tooth decay. While this selective approach would save costs, choosing a subset of families could stigmatize the program and lose the support of even those in need. On the other hand, oral hygiene is a personal and sensitive issue. Parents may have defensive, unfavorable views of the program and report it is not needed.

This quality improvement project is being rigorously evaluated. It assesses impacts on the member-participants as well as costs. The results of the evaluation will be used to determine if the complex intervention with intensive instruction and social support is needed or could be eliminated. In addition, the evaluation will inform if the program should be sustained and expanded. If proven effective, this simple intervention can be sustained by the dental managed care organization and replicated by other organizations and government.

## Abbreviations

CONSORT: Consolidated Standards of Reporting Trials
